# Institutional Determinants of Informal Payments for Health Services: An Exploratory Analysis across 117 Countries

**DOI:** 10.3390/ijerph182312421

**Published:** 2021-11-25

**Authors:** Cristian Incaltarau, Adrian V. Horodnic, Colin C. Williams, Liviu Oprea

**Affiliations:** 1 Centre for European Studies, Faculty of Law, “Alexandru Ioan Cuza” University of Iași, 700506 Iași, Romania; cristian.incaltarau@uaic.ro; 2 Faculty of Medicine, “Grigore T. Popa” University of Medicine and Pharmacy, 700115 Iași, Romania; liviu.oprea@umfiasi.ro; 3 Management School, University of Sheffield, Sheffield S10 2TN, UK; c.c.williams@sheffield.ac.uk

**Keywords:** informal payments, formal institutions, informal institutions, healthcare system, corruption, trust

## Abstract

Healthcare accessibility and equity remain important issues, as corruption in the form of informal payments is still prevalent in many countries across the world. This study employs a panel data analysis over the 2006–2013 period to explore the role of different institutional factors in explaining the prevalence of informal payments. Covering 117 countries, our findings confirm the significant role of both formal and informal institutions. Good governance, a higher trust among individuals, and a higher commitment to tackling corruption are associated with diminishing informal payments. In addition, higher shares of private finance, such as out-of-pocket and domestic private health expenditure, are also correlated with a lower prevalence of informal payments. In policy terms, this displays how correcting institutional imperfections may be among the most efficient ways to tackle informal payments in healthcare.

## 1. Introduction

The issue of good health has become more salient than ever amidst the current Covid-19 pandemic. Its magnitude and diffusion have caused major disruptions to the health systems [1,2]. The reallocation of health resources in dealing with the pandemic has reduced the capacity of providers to address other health issues, leading to accessibility constraints [3]. As a result, patients have tried to gain preferential access to medical services using different practices, including informal payments [4,5], which puts into question the efficiency and equity of health care provision. What is more, it seems that no progress has yet been made in fighting against informal payments, as these seem to be increasing. According to the Global Corruption Barometer (GCB) carried out by Transparency International, informal payments for medical services have almost doubled between 2006 and 2013, reaching 17% (of all respondents). Furthermore, healthcare also seems to be among the most affected sectors. For instance, in 2020, the medical sector reported the highest rate of informal payments, used in the form of bribes (6% of all respondents) [6].

The growth of informal payments raises concerns about achieving universal health coverage. The emergence of informal payments is clearly a sign that a health system fails to provide equitable access to healthcare, and the drivers behind such practices require particular attention. Unless the issues driving under-the-table payments are addressed, such informal payments will not be solved. Previous studies have emphasized the fundamental role played by both formal and informal institutions as drivers of informal payments. The formal institutional factors particularly relate to poor governance (e.g., poor regulations, lack of political stability) and institutional imperfections in providing healthcare services (e.g., resource efficiency, health accessibility; see [7,8,9,10]). From this perspective, informal payments may serve to help patients to access faster, better, or additional services [11,12]. Informal payments may also occur due to other informal institutional, namely socio-cultural, reasons, which might make patients more prone to such behavior (e.g., the social custom of expressing gratitude through informal payments, a higher inclination towards illicit behavior, a lack of alignment between the formal and informal institutions) [13,14].

Studying the occurrence of informal payments is crucial in order to deter any additional barriers to healthcare accessibility, and rebuild the trust between patients and health workers. Grounded in the institutional theory which emphasises the importance of institutions in explaining social, economic, and political dynamics [15], our study looks at the role of both formal and informal institutions in explaining and tackling unofficial payments. Formal institutions are ‘the rules of the game’, and refer to the regulations and the organizational environment [15,16]. Meanwhile, informal institutions are the ‘socially shared rules, usually unwritten, that are created, communicated and enforced outside of officially sanctioned channels’ [17]. To achieve this, a panel data analysis is carried out over 117 countries during the 2006–2013 period for estimating the effects of various categories of drivers related to both formal and informal institutions (countries included in the analysis are listed in the Appendix A). To our knowledge, there is no previous study exploring the level of informal payments which covers such a wide range of countries and years.

The remainder of this paper is organized as follows. The next section scrutinizes the literature to gauge the relationship between the institutional environment and informal payments in healthcare and, consequently, the hypotheses to be tested. Each hypothesis is grounded in institutional theory based on the findings in previous studies, and tested using variables related to the quality of governance, formal institutional imperfections in providing healthcare services (i.e., resource allocation and accessibility to healthcare services), and the quality of informal institutions (i.e., patient‘s norms and values). The third section describes the methodology, and examines the geographical heterogeneity of informal payments and its links with institutional quality. The estimation and interpretation of results and discussions are addressed in the fourth and fifth sections, while the last section draws conclusions.

### Tackling Informal Payments for Healthcare: What Role for Institutions?

Formal institutional imperfections are generally related to the quality of governance and resource availability, which can reduce the quality of medical services and restrain access to healthcare. Previous studies have shown that institutional bottlenecks may lead to a higher propensity for informal payments. This practice is associated with a lack of trust in political authorities, and is more widespread in countries with higher corruption levels. A lack of preventive measures, and poor regulation and law enforcement are also associated with higher informal payments [18,19,20]:

**Hypothesis** **1 (H1).** *Quality of Governance* *: Informal payments in health are more likely to occur with ill-functioning formal institutions.*

Health resource availability and the way it is allocated are also decisive for healthcare delivery. When resources are scarce, patients may look to gain preferential access by relying on informal payments [21,22]. Informal payments also stem from the fact that medical staff are underpaid [23,24]. Lower levels of health expenditures may also lead to underfinancing and, thus, to a precariousness of health workers conditions (for instance, the austerity measures pursued during the Great Recession that did not spare healthcare expenditures [25,26]). Therefore, our next hypothesis states that:

**Hypothesis** **2 (H2).** *Resource allocation* *: Higher shares of financial resources/private financing reduce informal payments in health.*

**Hypothesis** **2a (H2a).** *The higher the share of health resources, the lower is the prevalence of informal payments in health.*

Financing the health system is among the main challenges faced by states in order to provide universal and sustained access to health delivery. When public budget allocations are not sufficient, private sources need to compensate for it. On the one hand, patients need to make direct payments to healthcare providers at the time of service use, also known as out-of-pocket payments [27]. Although this alternative financing may undermine the equity of the healthcare system, this is often associated with lower informal payments levels [28]. On the other hand, the insufficient supply of medical services may leave larger room for private health care providers, which is also correlated with a lower prevalence of informal payments [11,29]. Therefore, our hypothesis is:

**Hypothesis** **2b (H2b).** *The higher the share of private financing, the lower the prevalence of informal payments in health.*

The increasing demand for healthcare occurring with development has emphasized the shortage of health workers. For instance, the worldwide nursing shortage was estimated to reach 5.9 million nurses in 2018 [30]. Though the more developed countries have the resources to attract foreign health workers [31], this leaves most of the deficit concentrated in low- and lower-middle-income countries [30,32]. This leaves less-developed countries struggling to provide fair healthcare accessibility, which leads the population to seek access to medical services by informal payments [22,33,34]:

**Hypothesis** **3 (H3).** *Healthcare accessibility* *: higher accessibility rates reduce informal payments in health.*

Besides the quality of governance, and the organization and financing of the health systems, informal payments may also stem from cultural and social factors, as well as from the individual’s norms and values. For instance, a more tolerant public attitude on corruption and illicit behavior may hinder the fight against informal payments [11]. A lack of trust in institutions, including health systems, may also make informal payments more likely [35]. Other times, informal payments may be rooted in social customs of giving a gift to express gratitude [13,36,37]. We therefore assume that:

**Hypothesis** **4 (H4).** *Patients’ norms and values* *: Informal payments in health are more likely to occur with ‘bad’ informal institutions.*

The role of informal institutions is also particularly important in preventing medical workers to exploit their informational asymmetry advantage compared with patients. Thus, individual self-regulation and trust becomes particularly important in the patient–doctor relationship [38].

## 2. Materials and Methods

In order to measure the level of informal payments in the health sector, this study uses the results of the Global Corruption Barometer (GCB) carried out by Transparency International. Among the biggest surveys worldwide tracking public opinion on corruption, the survey also captures their direct experience with bribery, and their willingness to stop corruption.

The dependent variable is whether patients made extra informal payments apart from the official fees. This is based on their response to the question: ‘In your contact or contacts with the institutions have your or anyone living in your household paid a bribe in any form in the past 12 months?’ Amongst the institutions listed, we have considered the share of population answering ‘Yes’ for ‘Medical and health services’.

The main interest explanatory variables tested are related to the quality of both formal and informal institutions [39]. The quality of governance for testing H1 is measured through the World Bank Quality of Governance Index, which reflects the ‘traditions and institutions by which authority in a country is exercised’. World Health Organization variables, such as the share of health expenditure, and out-of-pocket and domestic private health expenditure, are used for evaluating resource allocation in health for testing H2. Similarly, the number of medical doctors per 10,000 inhabitants (the lower the figure, the lower the healthcare accessibility) is used for evaluating healthcare accessibility, needed for testing H3. Meanwhile, for capturing informal institutions to test H4, two explanatory variables are used from the database, aggregated from various sources by the Quality of Government Institute at the University of Gothenburg [40], namely: social trust, and the share of population who feel personally obliged to report an act of corruption. Both measures are used to evaluate the quality of informal institutions. Indeed, social trust is extensively used in the literature as a measure for informal institutions (e.g., [41], the share of the population who feel personally obliged to report corruption captures the morality of the citizens, similarly to considering it unacceptable and informing authorities when other citizens are cheating on taxes [42,43]). Details about the main interest explanatory variables are provided in Table A1 in Appendix A.

For estimating the role of institutions in explaining informal payments in the health sector, our study relies on a panel data model over the 2006–2013 period. Although the Global Corruption Barometer (GCB) was initiated in 2003, the question referring to informal payments in the health sector was initially included in 2006. Therefore, our main interest variable, which derives from the GCB, was collected over the 2006–2007, 2009–2011, and 2013 (the panel is unbalanced, with full observations available for only 30 countries). The most recent version of the GCB, collected in 2017, did not publish any data referring to this question. The 2021 updated version of the database collected by the Quality of Government Institute at the University of Gothenburg also only includes data up to 2013 [40].

The fixed effects specification was indicated as preferable by the Hausman test, the null hypothesis that all-time dummies are jointly null was also rejected. Thus, the following two-way fixed effects model specification was estimated:

(1) $$Informal\;payments_{i,t}=\alpha +\beta Institutions_{i,t}+\gamma X_{i,t}+\delta_{t}+\eta_{i}+\varepsilon_{i,t},$$

where $Informal\;payments_{i,t}$ captures the prevalence of informal payments in healthcare in country *i* in year *t*; α is the intercept; $Institutions_{i,t}$ is a measure of institutional quality; $\;X_{i,t-1}$ includes a set of explanatory variables; $\delta_{t}$ are sets of time-specific intercepts; $\eta_{i}$ are the unobserved country specific effects; and $\varepsilon_{i,t}$ are the error terms. The homoscedasticity assumption was rejected by the modified Wald test, following Greene [44]. The Wooldridge test [45] for serial autocorrelation could not be performed due to the unbalanced nature of our sample. However, the errors were clustered by countries in order to produce asymptotically valid inferences in the presence of both heteroscedasticity and serial correlation.

A similar cross-section specification of the model was implemented to estimate the relation between informal institutions and informal payments in health, given the lack of time data regarding the population social norms and values. However, informal institutions are hardly variable in a shorter interval such as the one covered by our panel analysis, which makes the cross-section estimator a reliable alternative. Socio-economic variables are also included as controls in the analysis (e.g., education, employment, GDP), similar to various studies evaluating informal economy [46,47,48], corruption, and informal payments in the health sector [49,50,51].

It is worth mentioning that five year-variables were used for the cross-section model over the 2008–2013 period. For the variables measuring informal institutions, as the QoG Standard Cross Section dataset also specifies, data from and around 2017 is included, with a ±3 years margin interval.

As a robustness check, censored regression models were also estimated, which allows control for restrictions in the range of the dependent variable. First, the doubly censored tobit estimator was used [52] was used. This uses a maximum likelihood estimation, and is based on a random-effects specification due to the problems occurring in fixed-effects specifications [53]. The dependent variable is doubly censored, with the limits close to the minimum and the maximum values observed in our sample considering the countries and period analyzed (the lower limit was set at 0.00, and the upper limit at 0.67). Furthermore, our results were shown to be robust to changes of censoring limits. Second, we run a model by the fractional probit estimator, which is generally preferred when estimating a fractional response variable [54,55].

## 3. Results

### 3.1. Data Description

An overview on the data capturing the incidence of informal payments reveals that, on average, over 15% percent of patients have been involved in such payments when coming in contact with an institution providing health or medical services. (We focus on the cross-sectional data, which provides a better reflection of the level of informal payments in healthcare, since they were computed as averages over the 2009–2013 period). Zooming in more on the data shows a high heterogeneity, with rates between 0% and 66%. According to the Global Corruption Barometer (GCB) carried out by Transparency International, Switzerland was the only country to report 0% informal payments (in 2006). However, the yearly average over the entire period is slightly higher (1.48%). There are seven countries, from different geographical areas, with an average incidence below 1%, namely Brunei, Denmark, Germany, Iceland, Norway, South Korea, and Uruguay. At the other end, lies Burundi and Azerbaijan with 66.4 and 50.9% of respondents declaring that they (or their household) made informal payments when accessing health services.

**Figure 1:**
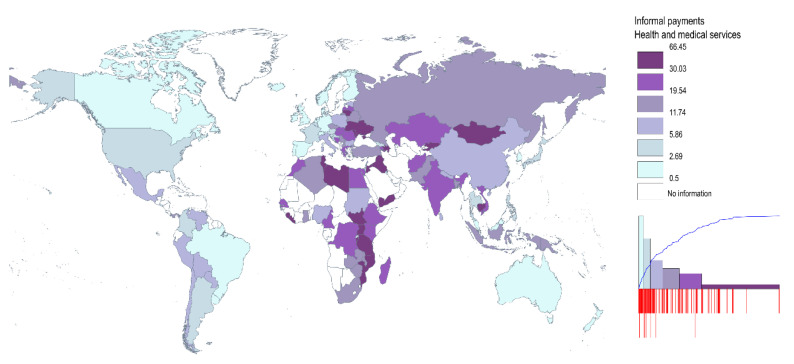
The incidence of under-the-table payments in health (% respondents, 2009–2013). Notes: This is based on their response to the question: ‘In your contact or contacts with the institutions have your or anyone living in your household paid a bribe in any form in the past 12 months?’; share of population answering ‘Yes’ for ‘Medical and health services’. The classes include an equal number of observations. Source: Authors’ representation using data from Transparency International.

It is also interesting to note that the incidence of under-the-table payments decreases with the development level: there is a 7% incidence in high income countries (following the World Bank classification), whereas informal payments spread to slightly over 20% in lower middle-income countries, and almost 31% in lower income countries (See Table A2 in Appendix A). Geographically, it is generally the countries from Africa, Middle-East, and Central and Southern Asia which display a higher incidence of informal payments. Informal payments are also spread among patients in Eastern Europe. Specifically, there are former Soviet states (Ukraine, Moldova, and Armenia), as well as EU member states, such as Lithuania, Slovakia, Romania, and Hungary, where this practice is more prevalent (See Figure 1). It is worth mentioning that though the classes include an equal number of observations, the amplitude of classes is different (see the histogram in Figure 1). Though the first two classes include a third of the countries represented, with values between 0 and 6%, the observations included in the last class have the highest standard deviation (including values between 66% and 30%).

**Figure 2:**
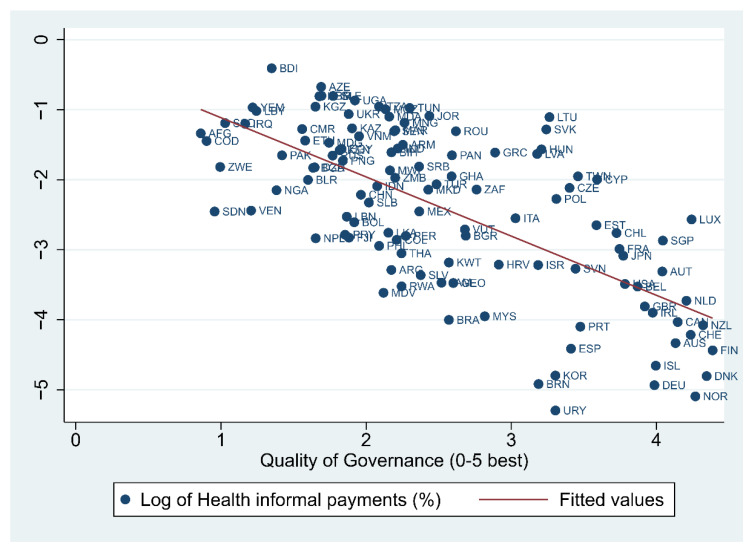
The relation between informal payments in health and the quality of governance. Note: Average values over the 2009–2013 period. Source: Authors’ representation using data from Transparency International and the World Bank.

**Figure A1:**
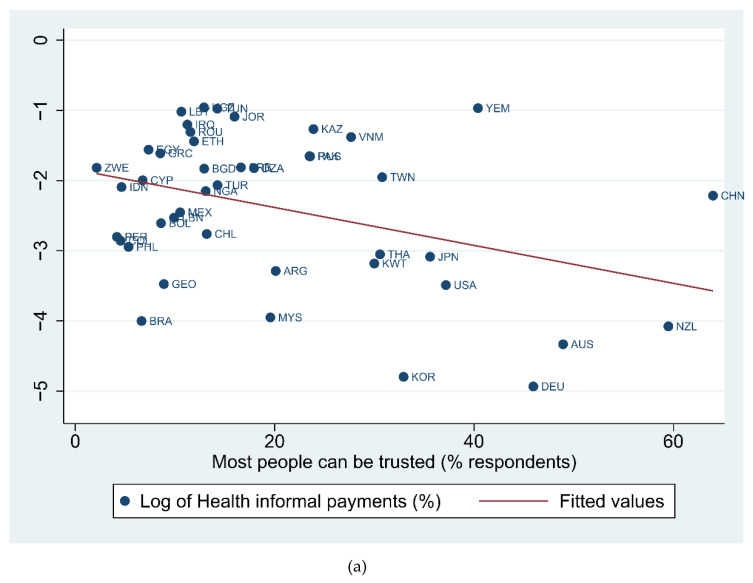

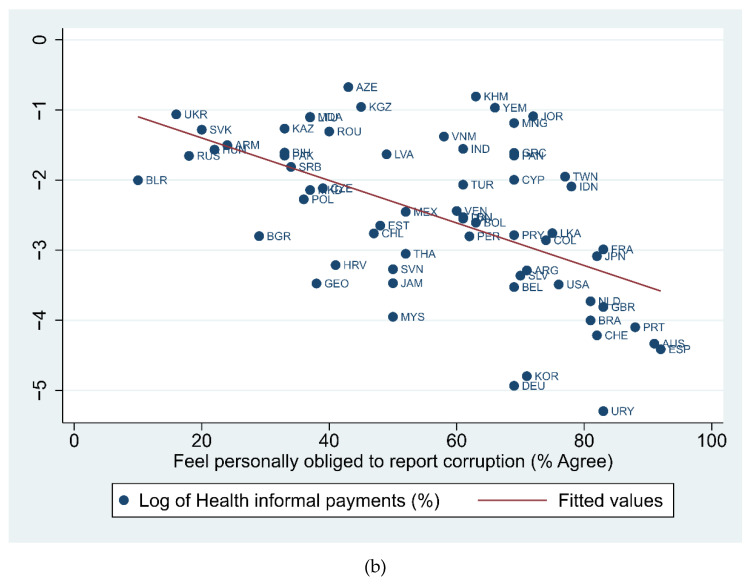
The relation between informal payments in health and informal institutions. (**a**) The relation between informal payments in health and social trust. *Source*: Authors’ representation using data from Transparency International and World Value Survey. (**b**) The relation between informal payments in health and the personal engagement in reporting corruption. *Source*: Authors’ representation using data from Transparency International.

Looking at the correlation between institutional quality and informal payments, this seems to be negatively associated. Countries with stronger institutions seem to generally display a lower incidence of informal healthcare payments (see Figure 2). The negative relation seems to hold when the strength of informal institutions is investigated. Stronger social capital, proxied by a higher trust in a population, seems to be associated with a lower incidence of under-the-table payments (Figure A1a in Appendix A). This is also true when looking at social norms, which indicates that informal payments for medical services are less widespread in countries where populations feel more engaged in reporting corruption (Figure A1b in Appendix A).

**Figure A2:**
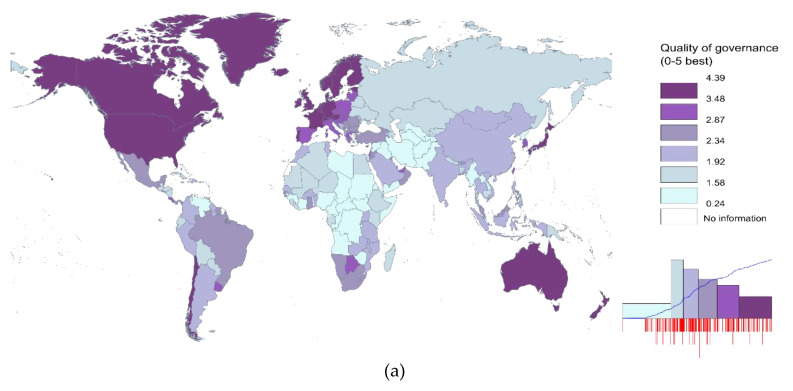

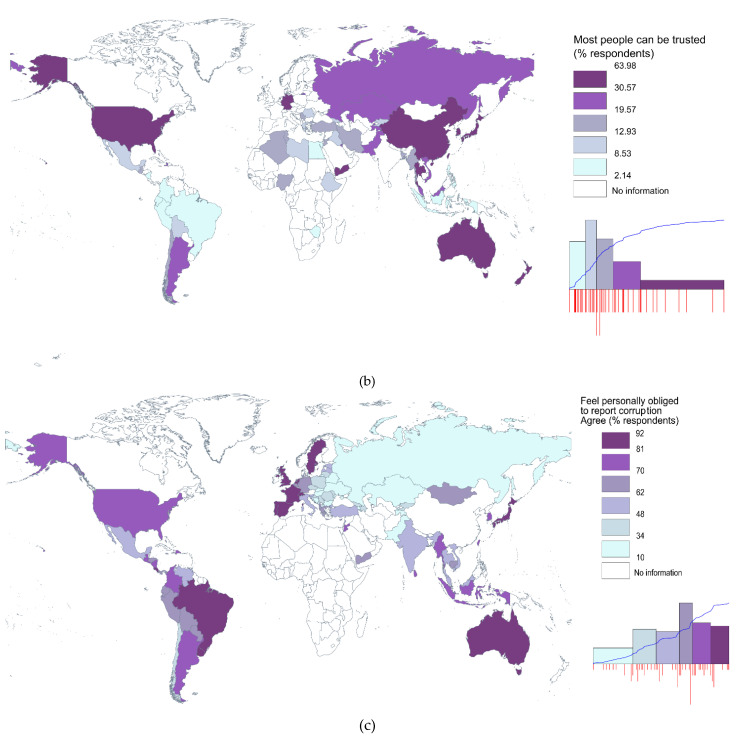
The quality of formal and informal institutions. (**a**) The quality of governance index (0–5 best, 2009–2013). *Notes*: The index was rescaled to 0–5 values. The six dimensions of the index were aggregated using a factorial analysis. The classes include an equal number of observations. *Source*: Authors’ representation using data from Transparency International and World Value Survey. (**b**) Social trust (% respondents). *Notes*: Share of people responding that ‘Most people can be trusted’ to the following question: ‘Generally speaking, would you say that most people can be trusted or that you need to be very careful in dealing with people?’ The values are from and around 2017, with a ±3 years margin interval. The classes include an equal number of observations. *Source*: Authors’ representation using data from the World Values Survey. (**c**) Personal engagement in reporting corruption (% respondents, 2009–2013). *Notes*: Share of people agreeing with the following statement: ‘Feel personally obliged to report corruption’. The values are from and around 2017, with a ±3 years margin interval. The classes include an equal number of observations. *Source*: Authors’ representation using data from Transparency International.

Though in terms of formal institutional strength, the higher and upper-middle income states generally display the higher scores (with the Scandinavian countries ranking on top—see Figure A2a in Appendix A), the relation with income level is less clear when it comes to informal institutions. For instance, it seems that there is also a quite high level of high social trust in low-income countries, such as Haiti, Tajikistan, or Yemen (Figure A2b in Appendix A). At the other end, there is a low level of trust in high income countries, such as Chile, Cyprus, and Greece. However, the population is also better engaged in reporting corruption in more developed countries, with around 90% percent of the population in Australia, Portugal, and Spain feeling personally obliged to report corruption acts (Figure A2c in Appendix A).

### 3.2. Findings

The estimation results of the role of institutional quality in tackling informal payments in healthcare are reported in Table 1 and Table 2: Table 1 displays results for the panel data model which captures the influence of formal institutions, and Table 2 accounts for the effects of informal institutions in a cross-sectional model. (The lack of times series data assessing the informal institutions worldwide forced us to reduce the panel model to a cross-sectional form). The panel data model includes both country and time fixed effects, which allows one to control for any country-specific characteristics unobserved in the model, or any unobserved time shocks. Due to its lower number of observations, the cross-section specification only includes income group dummies, following the World Bank income classification. This allows controlling for unobserved factors which are specific to different development levels.

Overall, our findings confirm that both formal and informal institutions play a significant role in reducing patients under-the-table payments. First, the coefficient of the quality of governance seems to be statistically significant and stable in most of the models, with values between 0.842 and −1.036 in both time dynamic and cross-section specifications (H1a is confirmed). The interpretation would suggest that a 1-point increase in the Quality of Governance Index (which corresponds to a 20% improvement, given the 0–5 scale) would reduce informal payments by 62% (specifically, the effect is computed as follows: ($e^{-0.978}-1$) × 100 = −62.4%) if the other variables are held constant (Model 2 in Table 1). The role of institutions in combat informal payments also holds when the censored estimators are used (columns 8–9 in Table 1 and Table 2). When computing elasticities (column 9 in Table 1), the results showed that under-the-table payments decrease when quality of governance improves at a rate such that, if the rate were constant, informal payments would decrease by 1.57% if quality of governance increased by 1%. The effect is stronger for the countries with lower quality of governance. For instance, for countries with a quality of governance of 1 (given the 0–5 scale), if the rest of the variables are at their corresponding values, a 1-point increase in quality of governance would lead to a decrease in informal payments by 0.10 percentage points. The effect decreases for countries with a quality of governance evaluated at 4, where the effect decreases to 0.03 percentage points. It is interesting to note that the coefficient displayed by the censored estimators is similar in the cross-sectional model (Table 2), thus, proving the robustness of our results.

The role of institutions in combating informal payments in healthcare does not seem to differ by the development level of countries. In order to test this hypothesis, we relied on several interaction terms. Specifically, for each of the institutional variables, we have also computed interaction terms with two binary variables accounting for the development level of countries. The two binary variables are taking the value one for the low income/high income countries, or zero otherwise (following the World Bank classification in 2018). As the interaction terms were not statistically significant, we decided not to report these results.

It is also interesting to note that the level of informal payments appears to be lower in health systems with higher shares of private financing. Larger shares of private expenditures, as well out-of-pocket shares, to current health expenditure seems to lead to lower informal payments, thus confirming Hypothesis 2.

When looking at accessibility to health resources, our results display that neither higher expenditures nor a higher density of medical workers make a significant contribution in tackling informal payments (infirming Hypothesis H2). Economic performance is not related to the incidence of informal payments, as both economic growth and GDP per capita are not statistically significant. However, bribes appear to be lower in countries with a higher level of urbanization and higher employment levels (It also worth mentioning that though in the panel specification employment level appears to be positively associated with informal payments (Table 1), the opposite is unveiled in the longitudinal models (Table 2). However, given the higher number of observations and the two-way fixed effects specification, we consider the first specification to be preferable and comment the results mainly according to them, where such contradictions may occur), which is generally also where more economically intensive activities are placed (Hypothesis 4 is partially confirmed). The higher propensity of informal payments in the urban areas may be explained by both the higher accessibility to healthcare services of the urban population, as well as by their higher income levels [49,56,57].

Second, the variables capturing the quality of informal institutions appear to also be negatively associated with a lower incidence of informal payments for healthcare, thus validating H1b. Of course, due to a lack of time series data, these variables could only be included in the cross-section model (Table 2). Our findings show that higher percentages of the population feeling personally obliged to report corruption also leads to a lower incidence of informal payments of patients (Model 2 in Table 2), which clearly hints that personal values play a leading role in illicit practices.

Aside from high quality institutions, a solid moral behavior and an increased social trust may also lead to lower informal payments. The two proxies for informal institutions are simultaneously significant in Model 6 (Table 2). However, when the quality of governance is also included in the model, it is only the variable referring to social trust which remains statistically significant. A 1% increase in the share of the population agreeing that most people can be trusted is associated with a 3.2% decrease in bribes paid by patients. However, it is worth mentioning that the informal payments variables do not turn statistically significant when the censored estimators are employed.

Finally, we do not find evidence for the role of education in mitigating illicit behavior, as the variable referring to the number of schooling years did only turn statistically significant in some of the cross-sectional models (Table 2). The same holds true for age dependency. Finally, if life expectancy appears to be positively associated with the level of informal payments when the two-way fixed effects is used, the opposite is true when running the censored estimations, which requires caution when interpreting this result.

## 4. Discussion

Our findings emphasize institutional conditions as an important prerequisite for tackling informal payments (a summary of the hypotheses tested is displayed in Table 3). Though a higher range and reach of health service provision may be attained by improving formal institutions, one also needs to enact a bottom-up approach in order to change the social norms and values.

The quality of health institutions is directly related to the general quality of government. Therefore, top-down policy initiatives should place greater emphasis on creating proper institutional conditions as a prerequisite for better healthcare and a lower incidence of informal payments. It is unlikely that the goals of efficient and equitable health care delivery can be achieved without addressing the formal institutional bottlenecks [58]. Our findings also show that positive externalities from economic growth might not be a sufficient condition for increasing health equity and reducing illicit behaviors, unless they are accompanied by solid institutions [59].

One of the main reasons behind informal payments is related to the fact that the health sector is not being provided sufficient funding [60,61,62]. This might drastically impact the accessibility and quality of care services by at least two mechanisms, both by the lack of necessary resources [63] and an unattractive wage level for health workers [64]. Both channels might increase migration propensity [65] and discourage younger people in embracing a medical career [66], which undermines the capacity of the health system to become self-sufficient [67]. It is also worth mentioning that increasing health funding and, consequently, the level of remuneration in the health sector, is not a sufficient condition either for improving healthcare delivery, or for reducing illicit behaviors, unless it is doubled by other reforms [8].

Particular attention is required during periods of economic turmoil. Previous studies have shown that during economic recession, there is an increased pressure on public budgets, which makes health systems more susceptible to financial cuts [68]. Therefore, attracting alternative private sources of finance might help health systems in facing future possible shocks. For instance, our results indicate that higher shares of private financing, such as out-of-pocket and domestic private health expenditure, are, overall, associated with a lower incidence of informal payments. On the one hand, supporting private providers of medical services might enlarge supply and, thus, accessibility for patients. It might also lead to an overall improvement of efficiency in delivering healthcare, by stepping up competition. Thus, widening private health coverage might be more efficient in mitigating informal payments and combating the moral hazard which occurs when extending the public health coverage [69]. On the other hand, increased attention is required when designing such policies, as the possible negative effects on vulnerable groups is already acknowledged in the literature [27,28]. Moreover, a greater reliance of the private market might also lead to opportunistic behaviors. In a sector where informational asymmetry is actually the rule, which makes patients vulnerable to opportunism and exploitation, policy-makers may want to focus on reducing the information disadvantage [38]. Therefore, a more attentive formulation of policy could be oriented towards increasing the motivation of health workers. Besides increasing the overall wage levels, providing bonuses or relying on a fees-for-services model instead of fixed salaries, or designing quality instead of quality-based criteria for reimbursement might increase their productivity and make them less willing to accept under-the-table payments [26].

In addition, raising accountability of the health personnel by basic monitoring tools, such as period internal assessments, might also reduce illicit payments, while also making medical service providers responsible if abuse occurs [29]. Other kinds of policy-initiatives, such as advertising campaigns, may help patients become aware that they are entitled to a second opinion before opting for a healthcare product. Advertising campaigns may also be helpful in making both patients and medical workers aware of the actual costs and systemic risks related to under-the-table payments. Pursuing educational campaigns might also be helpful in convincing citizens about their responsibility in paying taxes, in order to support public services, including healthcare [70].

Further developments of this study should also be considered. First, widening the geographical and time coverage of the data measuring informal payments may further test the validity of our findings. Though we have a better coverage regarding the quality of governance, the data measuring informal payments stop in 2013, and exclude many countries in Africa, Central America, and Central Asia. The unbalanced nature of our panel did not allow for such a specification. Finally, macroeconomic studies need to be complemented by more qualitative analysis which might better reflect more specific drivers explaining under-the-table payments. Where the majority of studies focus on the demand side of informal payments, the supply side, namely the health workers’ perspectives, also deserves more attention.

## 5. Conclusions

This study has focused on informal payments in the health sector which generate additional barriers to healthcare delivery, and erode the relation between patients and health workers. Specifically, the research has sought to explain informal payments through the lens of institutional theory. The role of formal institutions, as well as the population norms and values, was explored by carrying out a panel analysis. To our knowledge, there is no similar study employing a dynamic analysis which captures such a wide range of countries. The findings clearly emphasize that good governance, a higher trust among individuals, and a higher responsibility in tackling corruption are important prerequisites for tackling illicit payments. In addition, higher shares of private financing might also be helpful in fighting against informal payments, but a more attentive formulation of policies is required in order not to undermine the equity of healthcare services. This study provides a solid argument for the leading role of developing formal and informal institutions as a precondition for better healthcare. As previous studies argue [59], informal payments are actually just a symptom of an unfair treatment by the healthcare system. Reducing informal payments may primarily be achieved by a higher commitment to equity, and this can mainly be driven by good institutions.

## Figures and Tables

**Table 1:** Panel estimation of the role of formal institutions in explaining bribes in the health sector, 2006–2013.

	Two-Way Fixed Effects							Tobit	Fractional Probit
	(1)	(2)	(3)	(4)	(5)	(6)	(7)	(8)	(9)
Log of Education	−2.235	−2.518	−2.915	−2.636	−2.540	4.992	−7.302	−0.0183	−0.0542
	(4.709)	(4.822)	(4.875)	(4.975)	(4.528)	(5.431)	(4.653)	(0.0337)	(0.142)
Log of Age dependency	−1.282	−1.543	−1.596	−1.511	−1.569	−2.169	−2.402	−0.0604	−0.417 *
	(1.964)	(1.911)	(1.912)	(1.951)	(2.177)	(1.980)	(1.905)	(0.0581)	(0.249)
Log of Employment	2.546 **	3.001 **	2.977 **	3.006 **	3.059 **	3.280 ***	2.814 **	−0.0231	−0.256
	(1.280)	(1.225)	(1.214)	(1.229)	(1.171)	(1.240)	(1.138)	(0.0484)	(0.216)
Life expectancy	0.142 *	0.142 **	0.147 *	0.144 *	0.142 *	0.192 *	0.142 **	−0.00522 **	−0.0299 ***
	(0.0762)	(0.0687)	(0.0757)	(0.0764)	(0.0755)	(0.0991)	(0.0716)	(0.00233)	(0.0112)
Log of Health expenditures	−0.255	−0.180	−0.203	−0.0756	−0.0730	1.134 *	−0.245	0.0279	0.127
	(0.418)	(0.404)	(0.424)	(0.494)	(0.514)	(0.627)	(0.416)	(0.0252)	(0.105)
Log of Out-of-pocket expenditure	−0.660 **	−0.704 **							
	(0.287)	(0.272)							
Quality of governance		−0.978 *	−1.010 *	−1.024 *	−1.036 *	−0.499	−0.947 *	−0.0608 ***	−0.324 ***
		(0.550)	(0.554)	(0.563)	(0.535)	(0.631)	(0.529)	(0.0182)	(0.0839)
Log of Private health expenditure			−0.643 **	−0.696 **	−0.685 **	−1.126 **	−0.574 *	−0.0153	−0.0425
			(0.297)	(0.314)	(0.298)	(0.445)	(0.295)	(0.0178)	(0.0651)
GDP per capita growth				−0.00353					
				(0.00613)					
Log of GDP per capita					−0.0595				
					(0.699)				
Log of Doctors ratio						−0.257			
						(0.228)			
Urban							0.118 *		
							(0.0652)		
Obs./Countries	353/117	353/117	353/117	345/113	345/113	263/97	353/117	354/117	354/117
R-squared	0.223	0.233	0.230	0.231	0.230	0.306	0.240	-	-
BIC	525.3	526.4	528.1	529.3	529.6	366.5	529.2	−663.7	-

*Notes*: Robust standard errors, clustered by country, are given in parentheses. Significance levels: * *p* < 0.1, ** *p* < 0.05, *** *p* < 0.01. The dependent variable is in natural log, except for the tobit and fractional probit models. Time and country dummies are included, as well as a constant term, but the coefficients are not displayed in the table. The tobit model has a random effects specification, with the lower limit set at 0.00, and the upper limit at 0.67. BIC stands for the Bayesian information criterion. Source: Authors’ estimations.

**Table 2:** Cross-section estimation of the role of formal and informal institutions in explaining informal payments in health sector.

	OLS							Tobit	Fractional Probit
	(1)	(2)	(3)	(4)	(5)	(6)	(7)	(8)	(9)
Log of Education	0.540 *	0.563	0.478	0.833	0.901 *	0.831	0.991	0.184 ***	1.040 ***
	(0.290)	(0.652)	(0.515)	(0.604)	(0.449)	(0.749)	(0.690)	(0.0608)	(0.306)
Log of age dependency	−0.955 **	0.217	−0.886	0.0143	−0.334	−0.0428	0.174	−0.0315	0.191
	(0.474)	(0.962)	(0.770)	(0.975)	(0.710)	(1.241)	(1.209)	(0.0979)	(0.503)
Log of employment	−1.666 ***	−1.481 *	−2.353 ***	−1.513 *	−1.891 ***	−1.997 *	−1.830	−0.280 **	−1.554 ***
	(0.418)	(0.783)	(0.534)	(0.759)	(0.521)	(1.127)	(1.160)	(0.108)	(0.489)
Life expectancy	−0.0510 ***	−0.0857	−0.0442	−0.0619	0.0140	0.0110	0.0208	−0.00300	0.0238
	(0.0184)	(0.0660)	(0.0288)	(0.0604)	(0.0318)	(0.105)	(0.102)	(0.00856)	(0.0469)
Log of Health expenditure	−0.0272	−0.0786	−0.0530	−0.0594	−0.0390	−0.0348	−0.0379	−0.00811	−0.0886 *
	(0.0399)	(0.0688)	(0.0715)	(0.0633)	(0.0613)	(0.108)	(0.108)	(0.00653)	(0.0474)
Log of Private health expenditure	−0.00742	0.0139	−0.609	−0.0243	−0.491	−0.886	−0.807	−0.0673	−0.293
	(0.151)	(0.291)	(0.385)	(0.291)	(0.392)	(0.532)	(0.487)	(0.0404)	(0.192)
Quality of governance	−0.646 ***			−0.842 ***	−0.950 ***		−0.805	−0.0647 *	−0.404 **
	(0.186)			(0.298)	(0.337)		(0.500)	(0.0359)	(0.172)
Feel personally obliged to report corruption		−0.0161 **		−0.0108		−0.0219 *	−0.0166	−0.000390	−0.00486
		(0.00775)		(0.00838)		(0.0118)	(0.0144)	(0.00121)	(0.00545)
Trust			−0.0140		−0.0103	−0.0378 **	−0.0324 *	−0.000807	−0.00511
			(0.0131)		(0.0114)	(0.0168)	(0.0176)	(0.00124)	(0.00770)
Observations	113	63	42	63	42	28	28	29	29
BIC	296.6	178.0	118.9	173.5	114.2	85.46	85.45	−37.88	57.91
R-squared	0.584	0.539	0.583	0.598	0.659	0.649	0.689	-	-

*Notes*: The dependent variable is expressed in logs. Robust standard errors are given in parentheses. Significance levels: * *p* < 0.1, ** *p* < 0.05, *** *p* < 0.01. The dependent variable is in natural log, except for the tobit and fractional probit models. The lower limit for the tobit model was set at 0.00, and the upper limit at 0.67. Income level fixed effects following the World Bank classification (2018) are included, as well as a constant term, but the coefficients are not displayed in the table. BIC stands for the Bayesian information criterion. The variables are averaged over a 5-year period (2009–2013). The variables capturing informal institutions are from and around 2017, with a ±3 years margin interval.

**Table 3:** Summary of findings.

	Hypothesis	Result	
Formal Institutions	*H1. Quality of Governance:* Informal payments in health are more likely to occur with ill-functioning formal institutions.	Confirmed	Significant negative impact of quality of governance on informal payments.
	*H2. Resource allocation:* Higher shares of financial resources/private financing reduce informal payments in health.	Partially confirmed	
	*H2a.* The higher the share of health resources, the lower the prevalence of informal payments in health.	Not confirmed	Unsignificant impact of the share of health expenditures on informal payments in health.
	*H2b.* The higher the share of private financing, the lower the prevalence of informal payments in health.	Confirmed	Significant negative impact of out-of-pocket and domestic private health expenditure on informal payments in health.
	*H3. Healthcare accessibility*: higher accessibility rates reduce informal payments in health.	Not confirmed	Unsignificant impact of the ratio of physicians (per 10,000 inhabitants) on informal payments in health.
Informal Institutions	*H4. Patient’s norms and values*: Informal payments in health are more likely to occur with ‘bad’ informal institutions.	Confirmed	Significant negative impact of trust, and feel personally obliged to report corruption on informal payments in health.

## Data Availability

Publicly available datasets were analysed in this study. More details regarding the series used in the study are displayed in Table A1.

## Appendix A

List of countries included in our sample:

Afghanistan, Albania, Algeria, Argentina, Armenia, Australia, Austria, Azerbaijan, Burundi, Belgium, Bangladesh, Bulgaria, Bosnia and Herzegovina, Belarus, Bolivia, Brazil, Brunei, Canada, Chile, China, Cameroon, Congo, Democratic Republic of the Congo, Colombia, Cyprus, Czechia, Denmark, Dominican Republic, Egypt, Spain, Estonia, Ethiopia, Finland, Fiji, France, Gabon, Germany, Georgia, Ghana, Greece, Croatia, Hungary, Indonesia, India, Ireland, Iraq, Iceland, Israel, Italy, Jamaica, Jordan, Japan, Kazakhstan, Kenya, Kyrgyzstan, Cambodia, Korea, Republic of, Kuwait, Lebanon, Liberia, Sri Lanka, Lithuania, Luxembourg, Latvia, Morocco, Republic of Moldova, Madagascar, Maldives, Mexico, North Macedonia, Mongolia, Mozambique, Malawi, Malaysia, Nigeria, Netherlands, Norway, Nepal, New Zealand, Pakistan, Panama, Peru, Philippines, Papua New Guinea, Poland, Portugal, Paraguay, Romania, Russia, Rwanda, Senegal, Singapore, Solomon Islands, Sierra Leone, El Salvador, Serbia, South Sudan, Slovakia, Slovenia, Sweden, Switzerland, Thailand, Tunisia, Turkey, Tanzania, Uganda, Ukraine, Uruguay, United Kingdom, United States, Venezuela, Viet Nam, Vanuatu, Yemen, South Africa, Zambia, Zimbabwe.

**Table A1:** Data description and sources.

Variable	Description	Source
Informal payments		
Informal payments in health sector	Share of population responding ‘Yes’ when it comes to ‘Medical and health services’ to the following question: ‘In your contact or contacts with the institutions have your or anyone living in your household paid a bribe in any form in the past 12 months?’	The Global Corruption Barometer, Transparency InternationalDownloaded from QoG Standard dataset [40]
Formal institutions		
Quality of governance	The Quality of Governance Index (estimate). The index was rescaled to 0–5 (best) values. The six dimensions of the index were aggregated using a factorial analysis.	Worldwide Governance indicators database of World Bank [39]
Health expenditures	Current Health Expenditure (CHE) as % Gross Domestic Product (GDP), Percentage	World Health Organization
Out-of-pocket expenditure	Out-of-pocket (OOPS) as % of Current Health Expenditure (CHE), Percentage	World Health Organization
Private health expenditure	Domestic Private Health Expenditure (PVT-D) as % Current Health Expenditure (CHE), Percentage	World Health Organization
Doctor ratio	Medical doctors (per 10,000)	World Health Organization
Informal institutions		
Feel personally obliged to report corruption	Share of people agreeing with the following statement: ‘Feel personally obliged to report corruption’. The values are from and around 2017, with a ± 3 years margin interval.	The Global Corruption Barometer, Transparency InternationalDownloaded from QoG Standard dataset [40]
Trust	Share of people responding that ‘Most people can be trusted’ to the following question: ‘Generally speaking, would you say that most people can be trusted or that you need to be very careful in dealing with people?’ The values are from and around 2017, with a ± 3 years margin interval.	World Values SurveyDownloaded from QoG Standard dataset [40]
Other variables		
Age dependency	Age dependency ratio (% of working-age population)	World Bank
		
Education	Educational attainment (population weighted education per capita, age 25+, mean years)	Institute for Health Metrics and Evaluation [71]
Employment	Employment to population ratio, 15+, total (%) (modelled ILO estimate)	World Bank
GDP per capita	Real GDP per capita, PPP	PWT version 9.1 [72]
GDP per capita growth	Real GDP growth	
Life expectancy	Life expectancy at birth, total (years)	World Bank
Urban	Urban population (% of total population)	World Bank

**Table A2:** Descriptive statistics.

Variable	Obs.	Mean	Std. Dev.	Min	Max
Panel data					
Informal Payments					
Informal payments in health sector	354	13.39	14.02	0.00	66.45
High income	138	5.75	7.87	0.00	35.85
Upper-middle income	102	12.92	13.24	0.36	64.91
Lower-middle income	90	20.03	13.06	0.97	60.34
Low income	30	31.72	15.70	1.75	66.45
Formal institutions					
Quality of governance	354	2.66	0.94	0.79	4.43
Health expenditures	354	6.81	2.60	1.78	16.35
Out-of-pocket expenditure	354	33.20	18.62	1.20	82.88
Private health expenditure	354	41.19	19.00	1.20	84.60
Doctor ratio	264	25.77	18.00	0.13	78.09
Other variables					
Age dependency	354	56.60	15.91	26.99	104.70
Education	354	9.13	3.44	1.69	14.69
Employment	354	56.95	11.12	31.64	87.02
GDP per capita	346	21,318.13	18,789.94	746.30	91,533.16
GDP per capita growth	346	4.48	7.34	−27.87	36.08
Life expectancy	354	72.40	7.93	48.47	83.33
Urban	354	61.57	21.22	10.92	100.00
Cross-sectional data					
Informal payments					
Informal payments in health sector	113	15.00	13.68	0.50	66.45
High income	40	6.70	8.01	0.50	33.07
Upper-middle income	32	13.84	11.85	1.83	50.95
Lower-middle income	27	20.43	11.17	3.46	44.56
Low income	14	30.86	17.00	2.95	66.45
Formal institutions					
Quality of governance	113	2.55	0.93	0.86	4.39
Health expenditures	113	6.76	2.60	2.06	16.28
Private health expenditure	113	40.18	18.41	1.64	79.07
Informal institutions					
Feel personally obliged to report corruption	64	56.17	20.69	10.00	92.00
Trust	42	19.71	15.12	2.14	63.98
Other variables					
Age dependency	113	57.64	17.35	27.46	103.19
Education	113	8.88	3.60	1.73	14.46
Employment	113	57.61	12.18	33.49	86.46
Life expectancy	113	72.08	7.98	50.17	82.96

*Note*: Given that the data referring to informal payments in health is only available for 2006–2007, 2009–2011, and 2013, the variables are summarized for these periods. For cross-sectional variables, 5-year averages are computed (2009–2013). The variables from QoG Standard Cross Section dataset, and are from and around 2017, with a ±3 years margin interval (feel personally obliged to report corruption and trust). Income level is set following the World Bank classification (2018).
